# Pain mechanism and management strategy of rheumatoid arthritis

**DOI:** 10.3389/fpain.2025.1693399

**Published:** 2025-10-28

**Authors:** Dijun Wang, Ting Li, Weiqi Wang, Yonglan Ruan, Jiali Cai, Xiaojing Yan

**Affiliations:** ^1^ Changzhou Key Laboratory of Human Use Experience Research & Transformation of Menghe Medical School, Changzhou Hospital Affiliated to Nanjing University of Chinese Medicine, Changzhou, China; ^2^ Department of Neurology, Changzhou Hospital Affiliated to Nanjing University of Chinese Medicine, Changzhou, China

**Keywords:** pain, RA, neuroimmune interaction, therapy, inflammation

## Abstract

Rheumatoid arthritis (RA) pain is one of the most common forms of chronic pain in clinic. A large number of RA-related literature has been reported. At present, although some analgesic measures are used in clinic, pain management after drug treatment remains suboptimal in real-world settings, and clinically meaningful pain after treatment is still reported. RA pain is a complex pathological process that involves inflammatory response, neuroimmune interaction, peripheral and central nerve sensitization, autoantibodies, structural damage, and other dimensions. Although inflammatory reaction is the most common cause of RA-induced pain, neuroimmune interaction is the key and core of RA pain, and autoantibodies are one of the significant characteristics of RA, which can directly or indirectly lead to pain. In addition, joint structural damage is the final pathological stage and a serious consequence in the late stage of RA. This article aims to summarize the mechanisms of RA pain, which is helpful to further clarify the diagnosis and provide targeted treatment.

## 1 Introduction

Rheumatoid arthritis (RA) is a systemic autoimmune disease characterized by joint lesions (1) and is one of the most common chronic joint diseases. The pathologic process of RA includes autoimmune synovial inflammation and hyperplasia, autoantibody production, cartilage and bone destruction, and systemic features, including cardiovascular, pulmonary, psychological, and skeletal diseases (2, 3). Therefore, it is clinically called immortal cancer (4). The global incidence of RA is as high as 1% (5). The incidence of disability in RA is positively correlated with the duration of the disease. The longer the duration of the disease, the higher the incidence of disability and functional limitations. Epidemiologic data predict that the global population may exceed 31 million cases by mid-century, a growing trend that will pose a serious challenge to the global public health system (6).

The currently recognized pathogenesis of RA consists of two main pathways: genetic susceptibility and immune abnormalities (7). Genetic susceptibility is an important pathogenetic mechanism of RA. The HLA-DRB1 gene is the most widely studied gene in RA, and it presents guanylated autoantigens to activate T cells, triggering an abnormal immune response (8). Immunological abnormality is the core mechanism of RA. T cells recognize antigens in synovial tissue after non-specific inflammation and stimulate other cells to release the cytokine, which is responsible for eroding cartilage and bone (9, 10). Activation of B cells promotes abnormal production of autoimmune antibodies and secretion of cytokines (11). Macrophage activation further amplifies the Th17 response, leading to an increase in proinflammatory cytokines (12). In addition, environmental and microbial factors are also involved in the development of RA (13). For example, quinones in tobacco smoke induce protein citrullination (14), producing self-antigens. Silica particles activate NLRP3 inflammasome and promote interleukin (IL)-1β release (15, 16). Dysbiosis of gut microbiota can increase the load of citrulline antigen, further activating autoimmunity through molecular simulation mechanisms to trigger RA (17, 18).

Pain is the most thoughtful characteristic of RA, running through the entire pathological process of RA. In 1987, the American College of Rheumatology (ACR) first stated in its RA treatment guidelines that joint pain is an important diagnostic criterion for RA, and every subsequent version of the RA guidelines has repeatedly emphasized the importance of pain management in the RA treatment process. Up to 50% of RA patients suffer from chronic pain (19), 40% claim to frequently use opioids for pain relief (20), and more than 80% of RA patients seek medical attention for effective treatments to manage pain (21). Persistent joint pain is the core symptom of RA. In the early stage, pain is mainly caused by exudative inflammation, with abnormal exudation from the patient's joints, synovial tissues, and plasma skin, infiltration of inflammatory cells, and synovitis-induced swelling and pain in the small joints of the hands and feet as the first symptoms (22). In the progressive stage, non-inflammatory tumor-like cellular components from synovial tissues invade and destroy articular cartilage and paraprosthetic bone. This proliferative joint destruction leads to pain manifested as symmetric, polyarticular, and small-joint swelling and pain (23). In the middle and late stages, immune complexes (ICs) and complement infiltration may lead to primary non-inflammatory tissue necrosis, with granulation and fibrous tissue adhesions on the articular surfaces, resulting in fibrous joint ankylosis, which eventually progresses to generalized osseous ankylosis, with some patients experiencing cervical vertebrae, temporomandibular joints, and thoracoclavicular and acromioclavicular joints, which are associated with increased pain (24).

Pain severely impacts patients' quality of life, leading to extreme psychological problems such as depression and anxiety, increasing the economic burden on families and society, and closely correlating with the progression and prognosis of RA (25, 26). Therefore, pain relief is not only the ultimate demand of most patients but also an important criterion for evaluating the effectiveness of RA drug therapy. Pain management and preservation of physical function is the priority of RA treatment (27, 28).

Previous reports have suggested that RA pain is mainly inflammatory in nature, but in reality, the pain management of some patients after the use of anti-inflammatory RA drugs is not perfect. Therefore, summarizing RA pain mechanisms and seeking potential molecular mechanisms are the key issues and top priorities in pain management. In this paper, we will systematically elucidate the mechanism of action of RA pain from multiple dimensions, such as inflammatory response, immune-neural interaction, peripheral and central nerve sensitization, autoantibodies, and structural damage.

## 1.1 Mechanisms of pain

The International Association for the Study of Pain (IASP) defines pain as an unpleasant sensory and emotional experience associated with, or similar to, actual or potential tissue damage; pain is a subjective sensation. The mechanism of pain generation depends on its anatomical basis, including afferent pathways, the central nervous system (CNS), and efferent pathways (29).

The afferent pathway is responsible for pain perception, and the starting point of pain is the nociceptors, which can be functionally divided into three main categories: thermal nociceptors, mechanical nociceptors, and polymodal nociceptors (30). These receptors are found in the skin, joints, and muscles and are responsible for receiving external stimuli and converting them into electrical signals. Mechanical nociceptors are peripheral endings of mildly myelinated Aδ nerve fibers that are activated when sharp or intense pressure is perceived (31). Temperature nociceptors are peripheral endings of mildly myelinated Aδ nerve fibers that are activated when the temperature is higher than 45°C or lower than 5°C. Multiple nociceptors are peripheral endings of unmyelinated slow-conducting nerve fibers, C fibers, which may be activated when strong pressure, chemical, and temperature stimuli are perceived, where chemical stimuli include the inflammatory mediators such as prostaglandins (PGs), histamine, ILs, and tumor necrosis factor (TNF)-α (32).

Receptors are specialized sensory nerve endings that detect tissue damage or potential damage. Tissue damage and persistent inflammation activate pain receptors in the joints, which convert the stimulus into an electrical signal that is transmitted through the nerve via synapses in the posterior horn of the nerve (first-level neurons) to the posterior horn of the spinal cord, where it completes the transducer to the second level neurons to enter the transmission phase, which completes the conduction of nociception from the peripheral nervous system (PNS) to the CNS (32, 33). In this process, peripheral sensitization leads to primary nociceptive sensitization, which at the same time lowers the threshold of neuronal cell response to noxious stimuli and exacerbates the transmission and enhancement of pain signals (34–36).

Peripheral sensitization intensifies the excitability of CNS neurons, leading to central sensitization, which is the basis for the production of abnormal pain, including secondary nociceptive hypersensitivity and spontaneous pain. The nerve signals after peripheral sensitization are transmitted to the thalamus through the second level neurons, namely, the spinal thalamic tract which is mainly the white matter of the spinal cord (similar to a neural highway) in the spinal cord (32). The thalamus is an important relay station that senses the pain signal and maps that signal to the somatosensory cortex of the brain, which then localizes the site of pain. The cerebral cortex is responsible for recognizing the intensity, location, and nature of pain, while the limbic system is associated with emotional responses and pain perception (37).

**Figure 1:**
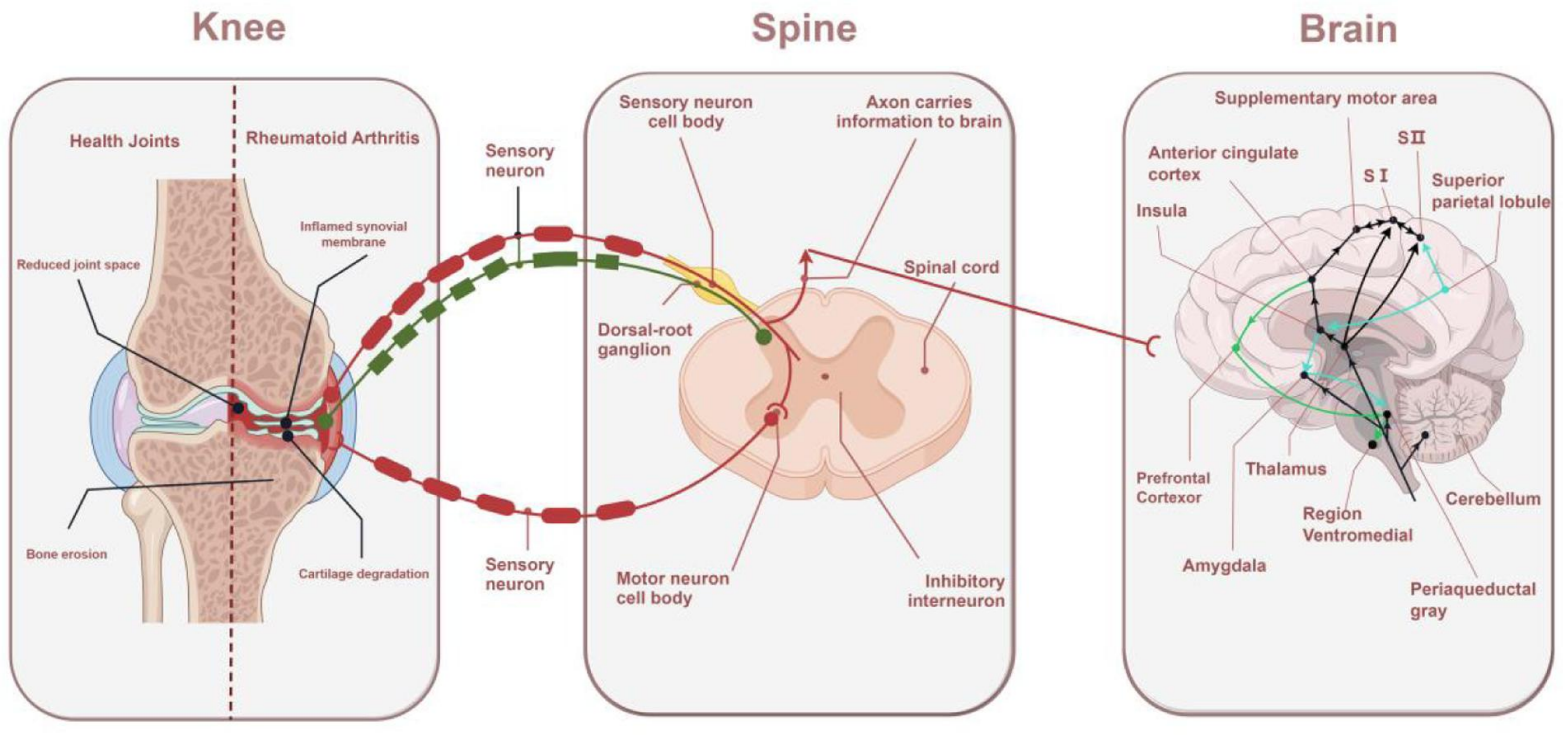
Mechanisms of RA pain: from the knee to the spinal cord to the brain. The sensory nerve endings in the joints sense pain and transmit signals to the spinal cord. The dorsal horn of the spinal cord amplifies the signals and transmits them to the brain, where different regions of the brain perceive pain and respond accordingly.

The transmission and perception of pain involves a complex network of multiple brain regions. Pain signals travel from the periphery to the spinal cord and then through the spinal thalamic tract to multiple regions of the brain for processing. Each region is responsible for a different function, from localizing the site of pain to modulating mood and attention to modulating pain perception through downstream pathways (38). These specific functional areas contribute differently to the perception of pain. The thalamus is an important relay station for pain signals and is responsible for transmitting pain signals from the periphery to several areas of the cerebral cortex for pain processing. The sensory cortex, located mainly in the parietal lobe of the brain, is responsible for the specific localization of pain. The amygdala is responsible for processing emotional responses associated with pain, such as fear, anxiety, and the emotional experience of pain (39). The rostral ventromedial medulla (RVM) has pain “on” and “off” cells that enhance or inhibit the transmission of pain signals by modulating the excitability of spinal dorsal horn neurons. The RVM is one of the central regions of pain modulation and is responsible for downward modulation of signals to the dorsal horn of the spinal cord (40). The periaqueductal gray (PAG) plays an important role in the inhibitory mechanisms of pain. The PAG activates the endogenous opioid system through a downstream pathway and releases endorphins which inhibit pain signaling and help individuals to reduce pain in the face of severe injury (41) (Figure 1).

## 2 RA pain and inflammatory response

The contribution of inflammation to RA pain is indisputable. Inflammatory response is the most common cause of RA pain. Most RA patients choose to seek medical attention due to joint swelling and pain, with clinical blood tests showing elevated systemic markers of inflammation, suggesting that the inflammatory response perhaps precedes the onset of pain (42). The severity of the inflammatory response is positively correlated with pain. In a clinical trial of patients with early-stage inflammatory RA, the mean pain visual analog scores (VAS 0–100) decreased by nearly 40 points in patients using the IL-6 inhibitor to lizumab, suggesting that anti-inflammatory therapy can effectively reduce the inflammatory pain response in RA (43). Although the mechanism of chronic generalized pain in patients is currently unclear, previous studies have provided some evidence.

### 2.1 Immune cells

The pathologic basis of RA is an abnormal immune response in synovial tissue. Immune cells and inflammatory mediators are central to the immune response. T cells, B cells, dendritic cells (DCs), and macrophages are deeply involved in RA pain. Th1/Th17 cells dominate the proinflammatory response. Th1 secretes interferon (IFN)-γ to activate macrophages, and Th17 secretes IL-17A/F and IL-22; recruits neutrophils; induces synovial fibroblasts to produce granulocyte–macrophage colony-stimulating factor (GM-CSF) and IL-6; and promotes RA inflammation amplification and pain exacerbation (44, 45). Treg cells have significant immunosuppressive functions, and their dysfunction is one of the potential mechanisms leading to the breakdown of self-tolerance in RA progression. Research has shown that the STAT3 pathway can inhibit Treg cells, leading to immune tolerance imbalance and the progression of RA (46). B cells differentiate into plasma cells after activation and produce autoantibodies such as RF and anticitrullinated protein antibody (ACPA). When the concentration of ICs is too high, it can trigger the activation of the complement system, lead to the formation of neutrophil extracellular traps (NETs), and directly combine with key players (including monocytes, osteoclasts, and osteoblasts) to promote the inflammatory response and mediate the bone destruction of joints in RA patients (47). Immature DCs absorb self-antigens such as ACPAs and migrate to lymph nodes. Mature DCs highly express MHC-II and co-stimulatory molecules (CD80/CD86), activate initial T cells, and secrete IL-12 to induce Th1 and IL-23 to maintain Th17 differentiation (48).

### 2.2 Inflammatory mediators

Inflammatory mediators are mainly divided into mediators released by cells and plasma-derived inflammatory mediators. Inflammatory mediators originating from cellular release include vasoactive amines such as arachidonic acids (thromboxanes, leukotrienes, and PG), cytokines (ILs, TNF-α, colony-stimulating factor, and interferon), and chemokines (49). These mediators are mainly produced or released by cells of various tissues and immune cells in response to stimulation or injury by damage factors. Plasma-derived inflammatory mediators include partially activated products formed by the activation of coagulation, fibrinolysis, kinin, and complement systems in plasma, including bradykinin, thrombin, and fibrin polypeptide. These systems are interrelated and together play a role in the inflammatory process. Excessive release of inflammatory mediators is a direct driver of pain. It is believed that tissue injury or inflammation leads to abnormal release of inflammatory mediators, which activate peripheral injury receptors and lower their excitatory threshold, leading to peripheral sensitization.

#### 2.2.1 Arachidic acid

PGs are derived from fatty acids within cell membranes. As important inflammatory mediators, they are responsible for maintaining homeostasis and mediating inflammation. PGs within the inflamed area are mainly derived from platelets and leukocytes and bind to their specific receptors, which are deeply involved in inflammation and pain formation. A clinical study showed that PGD2, PGE2, PGF2α, and 6-keto-F1α were significantly increased in the synovial fluid of patients with RA (50). PGE2, one of the major types of PG, is synthesized by the enzyme cyclooxygenase (COX), which mediates the major signs of RA inflammation, including edema, pain, swelling, and redness. Further studies have shown that large amounts of PGE2 produced and enriched in inflamed tissues can directly activate peripheral injury receptors, while indirectly stimulating aberrant expression of pain-related mediator substance P (SP) and calcitonin gene-related peptide (CGRP), which mediate the onset of pain (51). Therefore, non-steroidal anti-inflammatory drugs (NSAIDs), which inhibit COX activity and block the production and release of prostaglandin-like compounds, are currently one of the effective therapies for the treatment of RA pain (52). Expressed in basophils, mast cells, and monocytes as a potent chemotactic agent, leukotrienes bind to specific receptors on neutrophils and enhance neutrophil proliferation and migration. Most signs and symptoms of RA are mediated by recruiting leukocytes to initiate, coordinate, maintain, and amplify inflammatory responses (53).

As the most potent endogenous analgesic substance, the key role of bradykinin in RA pain should not be underestimated. It is a biological mediator of edema and can participate in the inflammatory process of RA through its specific receptor B2 (B2R). Previous studies have shown that activation of the peptide system leads to the release and overactivation of plasma kallikrein enzyme, which can directly stimulate the recruitment and migration of endothelial cells from the synovial membrane of arthritis which exacerbating RA symptoms in rats (54). Further studies showed that a B2R antagonist could alleviate the symptoms of arthritis induced by carrageenan and LPS, and significantly inhibit the pain of arthritis model animals (55). Bradykinin can promote IL-1 and TNF-induced PG biosynthesis in osteoblasts, including the levels of COX-2 and RANKL (2). These reports provide strong evidence for the involvement of bradykinin in RA pain.

#### 2.2.2 Cytokines

Cytokines play a central driving role in RA pain. After the occurrence of RA, chemokines rapidly recruit immune cells and release a large number of inflammatory factors, which are enriched at the joints and further aggravate the inflammation and pain (56, 57). A study showed that the use of the IL-1 receptor inhibitor AMG 108 in patients at the peak of acute inflammation significantly inhibited the expression of inflammatory mediators and attenuated allodynia (58). Involvement of the proinflammatory factors TNF-α and IL-6 is central to the pathogenesis of RA. TNF-α is secreted by macrophages and T cells and induces proliferation of synovial fibroblasts (formation of vascular opacities), activation of osteoclasts, and upregulation of other inflammatory factors (such as IL-1, IL-6). IL-6 is a pleiotropic cytokine that promotes B-cell differentiation and T-cell proliferation, induces acute-phase responses [e.g., elevated C-reactive protein (CRP)], and promotes Th17 differentiation through the STAT3 pathway. Recent studies have shown that other cytokines, such as IL-1, 17, 18, 23, and GM-CSF, also play a role (56, 59). It was found that symptoms such as morning stiffness in RA may be attributed to the circadian rhythm of plasma IL-1 concentrations which peak in the morning and evening and diminish during the day (60). IL-17 is involved in early and established RA, promoting the recruitment and activation of neutrophils, macrophages, and B cells. Meanwhile, IL-17 synergizes with TNF-α to activate the production of proinflammatory mediators, such as IL-1β, IL-6, IL-8, PGE2, and matrix metalloproteinases (MMPs), leading to structural destruction of the joints and promoting the progression of early inflammation to chronic arthritis (61). GM-CSF is secreted by T cells and endothelial cells and maintains neutrophil and macrophage survival, exacerbating synovium and pain (62). Therefore, newer biological disease-modifying antirheumatic drugs (DMARDs) are now based on cytokine blockers, and these biological DMARDs are effective in controlling inflammation and relieving severe pain in RA patients. A clinical study showed that relative to the placebo group, the TNF-α inhibitor adalimumab group could average significant changes in Health Assessment Questionnaire Disability Index (HAQ-DI) scores, DAS28-ESR Disease Activity scores, and VAS scores relative to baseline and that as many as 61.4% of patients claimed pain relief at week 24 (63). Despite the significant role of inflammation in RA pain, many randomized controlled trials have reported that medication significantly suppresses inflammation levels and reduces pain, but many patients still experience clinically significant levels of moderate pain after receiving therapy (64). Thus, in addition to the inflammatory response, other potential non-inflammatory molecular mechanisms are involved in RA pain.

## 3 Interaction between Ra pain and neuroimmunity

Neuroimmune interactions as a central mechanism of RA pain (65). The nervous system regulates the function of immune cells through signaling molecules such as neurotransmitters, neuropeptides, and hormones. The immune system influences neuronal activity through signaling molecules such as cytokines, chemokines, and inflammatory mediators, which interact with each other to mediate the production and maintenance of pain in RA (66).

### 3.1 Immune cell nervous system pathway

**Figure 2:**
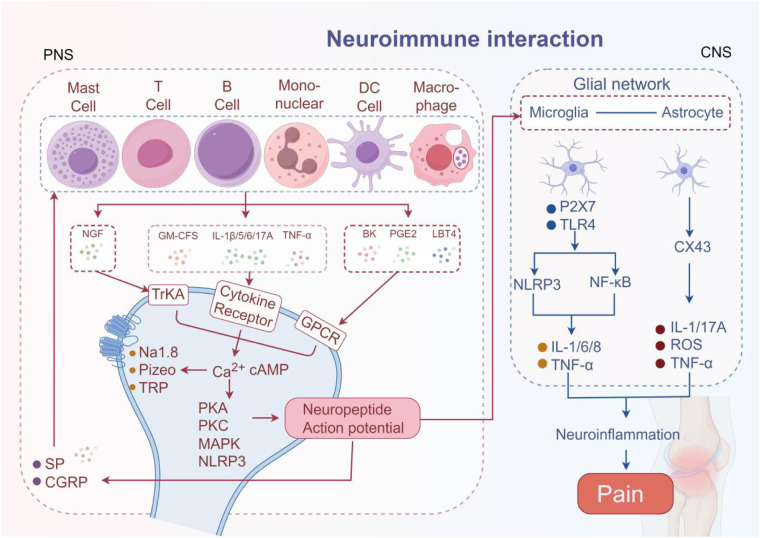
The neuroimmune interaction mechanism in RA pain. The pain mediators released by immune cells bind to corresponding receptors on neurons, activating neurons and transmitting pain signals. The activated neurons further release SP and CGRP to amplify pain signals. Simultaneously activate microglia and astrocytes, mediating pain.

Cytokines and chemokines secreted by immune cells act on neurons to affect the synthesis and release of neurotransmitters, thereby altering the function of the nervous system and participating in the regulation of pain. Sensory neurons are the core of the immune-neural pathway (67). Sensory neurons express a variety of receptors, including TNF-α receptors, IL receptors, transient receptor potential (TRP) channels, P2X channels, mechanical channels, G-protein-coupled receptors, and cytokine receptors, which allow direct detection of inflammatory mediators (68, 69). Sensory neurons can be categorized according to their function as injurious neurons, mechanosensory neurons, and thermosensory neurons, etc. Injurious neurons are particularly critical in immune regulation, forming close anatomical contacts with immune cells such as mast cells, dendritic cells, macrophages, T cells, B cells, and neutrophils, which mediate pain production (70–72). The above-mentioned key cell subpopulations are transported to the synovium, producing invasive tissue or vascular opacities, invasive proliferative vascular opacities, increased sensory nerve innervation, and loss of sympathetic nerve innervation in RA (73, 74). Mast cells mediate the release of IL-6, IL-8, TNF-α, histamine, SP, MIP-1α, and CGRP and stimulate monocytes to produce/release IL-1 family members and TNF-α, which sensitize sensory nerve endings and promote inflammation (75, 76). Activated macrophages accomplish pain signaling and amplification by producing IL-1β, IL-6, TNF-α, IL-12, and other cytokines that bind to specific receptors on injury receptors (77). T cells, a key element of adaptive immunity, are also strongly associated with pain. Following nerve injury, T cells infiltrate the dorsal root ganglia (DRG) and release the pro-nociceptive mediator leukocyte elastase, leading to mechanically abnormal pain (78). B cells in the synovium activate T cells and plasma cells indirectly through antigen presentation and promote the production of IL-21 and autoimmune antibodies, while directly secreting TNF-α, IL-6, IL-8, and CCL3 to stimulate sensory neurons and develop nociception (11). Currently recognized as the most effective antigen-presenting cells, DCs are the strongest helper of the immune system. When combined with T cells, they can secrete large amounts of IL-12, IL-18, and IFN-γ, contributing to the mediation of pain (79). More interestingly, recent studies have found that DC cells have a surprising effect on immunotherapy resistance (48) (Figure 2).

### 3.2 Neurotransmitter immune cell pathway

Neurotransmitters act directly on receptors on the surface of immune cells to regulate their function. Inflammatory responses in RA activate injury receptors that directly lead to peripheral sensitization. Acetylcholine (ACh), SP, and the CGRP are over-released at the site of injury, bind to neurotransmitter receptors on the surface of immune cells, and form a structure similar to the neural synapse that increases cytokine production (30). Neuropeptides not only cause vasodilatation, plasma extravasation, and inflammatory cell infiltration, but inflammatory mediators lower the threshold of injury receptors, further exacerbating the inflammatory response and peripheral sensitization, creating a vicious cycle of “peripheral sensitization.” Studies have shown that persistent peripheral nerve discharges for >72 h can trigger permanent changes in gene expression (80).

ACh is most widely known to be a neurotransmitter. Both neurons and immune cells (such as macrophages, T cells, B cells, and NK cells) can release acetylcholine. Studies have shown that activation of β2AR in early RA induces CD4T cells to release ACh, which mediates the migration of proinflammatory immune cells to target tissues (81).

At the joint level, CGRP acts on receptors expressed on synovial-resident cells, peripheral endings of sensory neurons, and inflammatory cells. CGRP can release proinflammatory mediators indirectly via macrophages or can directly activate sensory nerve fibers expressing CGRP receptors and pain signaling via protein kinase A (PKA) (82). It has been shown that CGRP inhibits T-cell activation by binding to the CLR/RAMP1 receptor complex and inhibiting dendritic cell maturation and MHC-II molecule expression, while CGRP promotes macrophage conversion to an M2 anti-inflammatory phenotype, inhibits IL-12 secretion, and enhances IL-10 production (83, 84).

SP is mainly distributed on C and Aδ nerve fibers and is released from sensory neuron endings by injurious stimuli. SP can chemotaxis immune cells and regulate cytokine production. SP has a chemotactic effect on T cells, monocyte macrophages, and eosinophils and acts as a chemokine for neutrophils, which directly affects their migration to the site of inflammation. It can also activate mast cells, causing intracellular Ca^2+^ mobilization and cell degranulation, leading to the release of histamine and protease. These processes exacerbate local vascular permeability and inflammatory infiltration, ultimately causing pain (85). SP also promotes the release of IL-1 and IL-8 from immune cells, the production and release of IL-1 and IL-6 from monocyte macrophages, and the production of IL-2 by activated T cells and the expression of IL-2 receptors. Previous studies have shown that SP can activate spinal nociceptive neurons by binding to immune cell neurokinin 1 receptor (NK1R) and activating the Mitogen-activated protein kinase (MAPK)/NF-κB pathway in macrophages, leading to excessive release of proinflammatory cytokines (e.g., IL-6 and TNF-α) (86–88). A clinical study of osteoarthritis (OA) and RA evaluating the relationship between pain intensity and serum SP concentrations in patients found that all patients had high serum concentrations of SP and that serum SP levels were significantly higher in patients with RA than in patients with OA. High serum SP levels are one of the important pathogenic mechanisms of RA pain (89).

### 3.3 Neurohormone immune cell pathway

Neurohormones such as norepinephrine (NA) and nerve growth factor (NGF) secreted by the nervous system can act on receptors on the surface of immune cells to regulate their differentiation, activation, and inflammatory response. NGF is a key mediator of inflammation and pain, and the maintenance of persistent inflammatory pain is a major biological effect of NGF. NGF is enriched in the synovial (89, 90) and osteochondral junctions of patients with RA and exerts its proinflammatory effects through a variety of mechanisms, including mast-cell sensitization and an increase in the synthesis and release of neuropeptides (CGRP and SP), which lead to vasodilation and plasma extravasation, triggering painful behavior (91). Elevated levels of NGF after organismal injury promote the growth of injured nerve fibers (C fibers) and enhance pain signaling through TrkA receptors, which are high-affinity signaling receptors for a variety of neurotrophic factors such as NGF, brain-derived neurotrophic factor (BDNF), or neurotrophin-3,4 (92).

Norepinephrine is the main neurohormone of the sympathetic nervous system, and large amounts of it are released from sympathetic nerve endings which can act either directly on injurious receptors or indirectly on α- and β-adrenergic receptors on immune cells to cause increased sensitivity of injurious receptors (93). It has been shown that NA inhibits TNF-α secretion from macrophages via β2-adrenergic receptors, resulting in negative feedback regulation. Macrophage and T-cell infiltration releases proinflammatory factors such as TNF-α and IL-17, which activate nerve endings and further exacerbate the inflammatory response and peripheral sensitization. Disease activity in RA is significantly correlated with circulating levels of IL-17A produced by T cells, which upregulates proinflammatory genes through activation of NF-κB, MAPK, and the C/EBP cascade response (94).

A complex neuroimmune–endocrine network is involved in RA pain. The hypothalamic–pituitary–adrenal (HPA) axis is a key component of the neuroendocrine system that has been shown to regulate the secretion of proinflammatory cytokines. It is responsible for maintaining homeostasis and ensuring that hormone levels, such as endogenous glucocorticoids (GCs), are maintained within normal ranges (95) and is thought to be an important regulator of the onset and progression of RA pain (96). The HPA axis function is severely dysfunctional in RA patients (97), which can lead to elevated cortisol levels and suppression of immune system function, thereby exacerbating the inflammatory response and pain perception in RA patients. In an adjuvant-induced model of arthritis (AIA), significant increases in cytokines (including IL-1, IL-6 in the pituitary gland and spleen, IL-6, and IL-1β) have been found after the adrenal glands of the animals have been removed (98). One study found that physiologic GCs broadly modulate the inflammatory process in RA by administering glucocorticoid (GS) receptor antagonists that can reverse P-selectin-mediated rolling responses. Inadequate physiologic GS response renders inflammation susceptible or persistent/immune cell activation by reducing autoantigen load (99). One study showed that IL-6 levels in RA patients were inversely correlated with cortisol levels, which has led to the recommendation that cortisol levels be used as one of the criteria for judging functioning in RA patients (100, 101).

### 3.4 Neuroglial cell-mediated neuroimmune interactions

Glial cells play an important role in the occurrence and development of chronic pain. Activated glial cells can release proinflammatory cytokines, thereby amplifying local and central inflammatory responses and exacerbating changes in the nervous system. Simultaneously secreting various neurotransmitters, affecting the excitability of neurons, forming a vicious cycle, and continuously maintaining a chronic pain state.

Central sensitization is a key part of pain (102). Continuous nociceptive stimulation leads to increased excitability of spinal dorsal horn neurons and excessive activation of N-methyl-D-aspartate receptors (NMDA), resulting in changes in synaptic plasticity. Subsequently, glial cells, mainly microglia and astrocytes, release inflammatory factors and excitatory amino acids, IL-1β, BDNF, and other mediators after activation, causing increased neuronal excitability and synaptic plasticity and promoting synaptic remodeling and pain signal amplification (103). Clinical studies have shown that the area of mechanical nociceptive hypersensitivity in centrally sensitized patients can exceed the site of primary injury by more than three times. Finally, patients with RA have a reduced function of the downstream inhibitory system, which leads to reduced inhibition of injurious information in the dorsal horn of the spinal cord, exacerbating pain perception (78).

Glial cells are round-the-clock partners of neurons, and cytokine expression is significantly increased in activated astrocytes and microglia during arthritis-induced hypersensitivity reactions (104). Collagen-induced arthritis (CIA) anti-TNF therapy exerts analgesic effects similar to COX2 by decreasing glial cell activity (105). The neuroglial inhibitor hexoketone cacodylate effectively relieves pain and hypersensitivity induced by advanced collagen antibody induced arthritis (CAIA), suggesting that microglia and astrocytes are potential targets for anti-RA pain (106).

Microglia and astrocytes are functional cells for central sensitization in the dorsal horn of the spinal cord and are activated to release inflammatory factors, chemokines, and neurotransmitters, which promote changes in neuronal excitability and synaptic plasticity, contributing to central sensitization and the generation and maintenance of chronic pain. Microglia are resident immune effector cells in the CNS, accounting for approximately 20% of the total number of glial cells in the CNS (103), and are rapidly activated and recruited to the site of injury after injury occurs (107). Subsequently, a variety of pro-injury mediators are synthesized and released, including cytokines TNF and IL-1β, chemokines CC chemokines (CCs), CXC chemokines (CXCs), reactive oxygen species (ROS), NGF, and NO, which are essential in RA pain (108–111).

Astrocytes are the most widely distributed class of cells in the mammalian brain and the largest of the glial cells, which express a variety of neurotransmitter receptors [SP, CGRP, γ-aminobutyric acid (GABA) receptors] (112). The dorsal horn of the spinal cord after peripheral nerve injury excessively releases a variety of transmitters that bind to specific receptors on astrocytes, thereby activating astrocytes to produce and secrete a variety of neurotransmitters (such as NMDA and NGF) involved in the maintenance of pain (113, 114). Rapid increase in the expression of Glial fibrillary acidic protein (GFAP), a marker of astrocyte activation, in the spinal cord of CIA model rats (115). The mRNA and protein levels of inflammatory mediators such as IL-1β, IL-6, and TNF-α were significantly elevated in the spinal cord of AIA rats. A significant increase in the number of astrocytes was found after 10 days. The activated astrocytes led to a dysfunction of the downstream inhibitory system, which further led to a weakened inhibition of injurious messages in the dorsal horn of the spinal cord, thus exacerbating RA pain perception.

Interestingly, in chronic pain models of nerve injury or arthritis, although activation occurred significantly earlier in microglia than in astrocytes, the role of astrocytes in maintaining the duration of chronic pain was more pronounced than that of microglia, and the astrocyte response was more sustained than the microglial response. Therefore, it is hypothesized that astrocyte activation is primarily associated with long-term chronic pain in RA, whereas microglia are more specialized in acute pain or the pre-existing phase of chronic pain in RA (116–118).

## 4 Autoantibody and immune complex deposition of ACPA and RF

Autoantibodies are a common feature of RA patients, with studies estimating that >50% of RA patients carry autoantibodies, and the degree of autoantibody response is a key determinant of RA progression and one of the important mechanisms of pain in RA patients (119). The Rheumatism Alliance Standard [ACR/European Alliance of Associations for Rheumatology (EULAR)] has repeatedly emphasized the important weight of autoantibody ACPAs and RF in the classification of RA (120).

Specific autoantibodies complex with collagen at the joints and trigger inflammation. ACPA, also known as anti-citrullinated peptide (CCP) antibody, is a polypeptide fragment of cyclic polyfilament proteins. ACPA recognizes antigens such as citrullinated fibrinogen and poikilocyte protein to form ICs, which activate the monocyte, macrophage, and complement systems via the Fcγ receptor. Complement C5a chemotaxis neutrophils to the synovial membrane, releasing NETs, further exposing citrullinated antigens, and forming a positive feedback loop leading to the development of joint pain (121, 122). Reports indicate that approximately 60% of patients with early RA and approximately 80% of patients with confirmed RA are positive for ACPA in peripheral serum and that an abnormal increase in pathogenic ACPA plays a critical role in the development of RA pain and immune maintenance (123, 124). Interestingly, ACPA is not a specific feature of all traditional animal models of RA, and the direct role of ACPA is as a disease mediator rather than a serological marker (125, 126). The synovial and subcutaneous tissues of RA patients abnormally produce large amounts of ACPA (127). Compared with ACPA-negative patients, ACPA-positive RA patients exhibit more severe inflammatory infiltration and joint bone erosion, increased disease activity, significantly increased incidence of pain, and poorer prognosis (128, 129). The appearance of pain in RA prior to inflammation of the joints and following anti-inflammatory treatment has been associated with autoantibodies. Wigerblad et al. (130) administered ACPA to mice and found that ACPA prolonged injury-perceiving behavior and that this pain behavior was still observed after anti-inflammatory treatment. They hypothesized that this was due to ACPA stimulation of mouse osteoclasts leading to osteoclast activation and chemokine CXCL1 recruitment, which resulted in the release of IL-8. It has been suggested that ACPA binds to osteoclast surface receptors and then activates the differentiation of osteoclast precursors and influences osteoclasts *in vitro* and *in vivo*, leading to the formation of bone erosion and the development of pain. During this process, ACPA initiates an autoinflammatory response via the Fc receptor and complement cascade reactions. Based on the Fc-mediated mechanism, injection of IgG-ICs into joints induces acute joint pain that is not accompanied by significant joint inflammation (131, 132). ACPA also acts on immune cells to exacerbate inflammatory cell infiltration and can drive macrophage cytokine production and exacerbate pain production by co-stimulating FcγIIa via TLR4, which activates Fcγ receptors on macrophages to accelerate TNF-α secretion (133). It can also activate monocytes by binding to GRP78 cell surface receptors, which drives NF-κB activation and cytokine production (134).

RF is an autoantibody that targets the Fc fragment of denatured IgG and is the first autoantibody described in RA. It is one of the characteristic autoantibodies of RA (135), approximately 75% of RA patients have positive serum RF (136), and 85% of patients test positive in the first 2 years of onset. Due to the slow fluctuation of serum RF levels, it is currently not possible to use RF to track the progression of RA (137). However, RF is crucial in the assessment and treatment of RA pain. RF deposits in synovium and vascular walls, activating the classical complement pathway, leading to local tissue damage and pain (138). Clinically, it is believed that patients with high titers of IgM type RF in their serum have more severe joint lesions than those with negative serum RF (139). As the most common antibodies in RA, ACPA and RF appear to act independently, but in reality, they often interact with each other simultaneously. ACPA levels and RF status are independently correlated with the progression of RA, and patients with high levels of positive RF have a higher risk of experiencing clinical symptoms such as pain (140). Interestingly, in another clinical study, the proportion of RA patients who experienced a transition from serum ACPA negative to ACPA positive was 5.4%. When ACPA-negative RA patients are classified according to their RF status, only the serum of the RF-positive subgroup is converted to ACPA-positive (141). The pain in RF and ACPA-positive patients may be attributed to the fact that RF exacerbates bone loss and destruction in ACPA-positive patients (142).

## 5 Structural damage and mechanical pain

RA is a multicompartmental disease involving not only multiple joints but also bone marrow, synovium, cartilage, and muscle tissue (143). Bone structure damage is the key to RA pain. Inflammatory cells, cytokines, and abnormal immune conditions disrupt the balance between local osteoblasts and osteoclasts, and osteoclast bone resorption induces the loss of bone parenchyma and trabeculae, leading to localized osteoporosis, bone destruction, and the development of pain (144, 145). In addition, decreased physical activity and medications such as steroids after RA diagnosis can exacerbate systemic osteoporosis and pain in patients (146).

The osteochondral junction is the source of pain in RA (147). Proinflammatory cytokines such as TNF-α, IL-6, and IL-17 promote osteoclast differentiation by inhibiting osteoclast activity and modulating the RANKL/OPG axis dysregulation. Meanwhile, ACPAs directly bind to guanylylated proteins on osteoclast precursors to enhance their resorption. These pathological processes lead to bone erosion, cartilage damage, joint destruction, and dyskinesia. Cartilage destruction and bone erosion lead to rough joint surfaces and increased friction during movement, and severe bone erosion increases the exposure of nerve endings, which directly stimulate pain receptors such as TRP, Piezo, and Na1.8 to trigger pain behaviors (123, 124, 148). At the same time, protons secreted by osteoclasts create an acidic environment that activates acid-sensing ion channels (ASICs) on sensory neurons (149). In addition, ligamentous laxity and joint structural instability in some RA patients lead to abnormal load distribution, which triggers repetitive microtrauma, which in turn activates injury receptors and directly triggers pain (150). CT scans of RA patients with knee osteoarthritis show loss and breakage of bone trabeculae. There is a highly significant correlation between the patient's pain and the degree of joint destruction; therefore, amelioration of joint destruction and restoration of joint homeostasis are key to RA pain management (151). In clinical practice, the administration of naproxen can significantly prolong the humane endpoint time of RA patients and improve their health score, pain level, and trabecular thickness, reducing abnormal mechanical pain and cold pain threshold (175). Strict control of RA disease activity through drug therapy can effectively prevent and stabilize bone erosion, bone destruction, joint stiffness, and secondary osteoarthritis, but its ability to significantly improve systemic bone loss and osteoporosis is limited.

Although the specific mechanism of joint destruction in RA patients is unclear, some potential mechanisms have been discovered and proposed. MMPs can degrade various protein components in the extracellular matrix ECM, and fibroblast-like synovial cells (FLS) are the main effectors of their degradation in RA (152). MMPs are involved in processes such as osteoblast differentiation, bone formation, bone resorption, recruitment, and migration of osteoclasts. The pathological process related to bone remodeling that leads to bone resorption or formation imbalance in RA is caused by overexpression of MMPs and abnormal changes in ECM (153). MMPs can directly act on osteoclast precursors, inducing their proliferation and differentiation, as well as directly stimulating the activation of mature osteoclasts, leading to abnormal increases in GM-CSF, IL-6, IL-11, IL-1, and TNF-α. These increased mediators further promote the breakdown metabolism of synovial cartilage, resulting in typical periarticular bone erosion (24). RANKL is an osteoclast differentiation factor that activates innate immune cells to produce proinflammatory cytokines, collectively promoting osteoclast formation. The expression of RANKL in the synovial tissue of RA patients is increased (154). Previous studies have shown that CXCL16 upregulates the expression of RANKL in RA-FLS through the JAK2/STAT3 and p38/MAPK signaling pathways (155).

**Figure 3:**
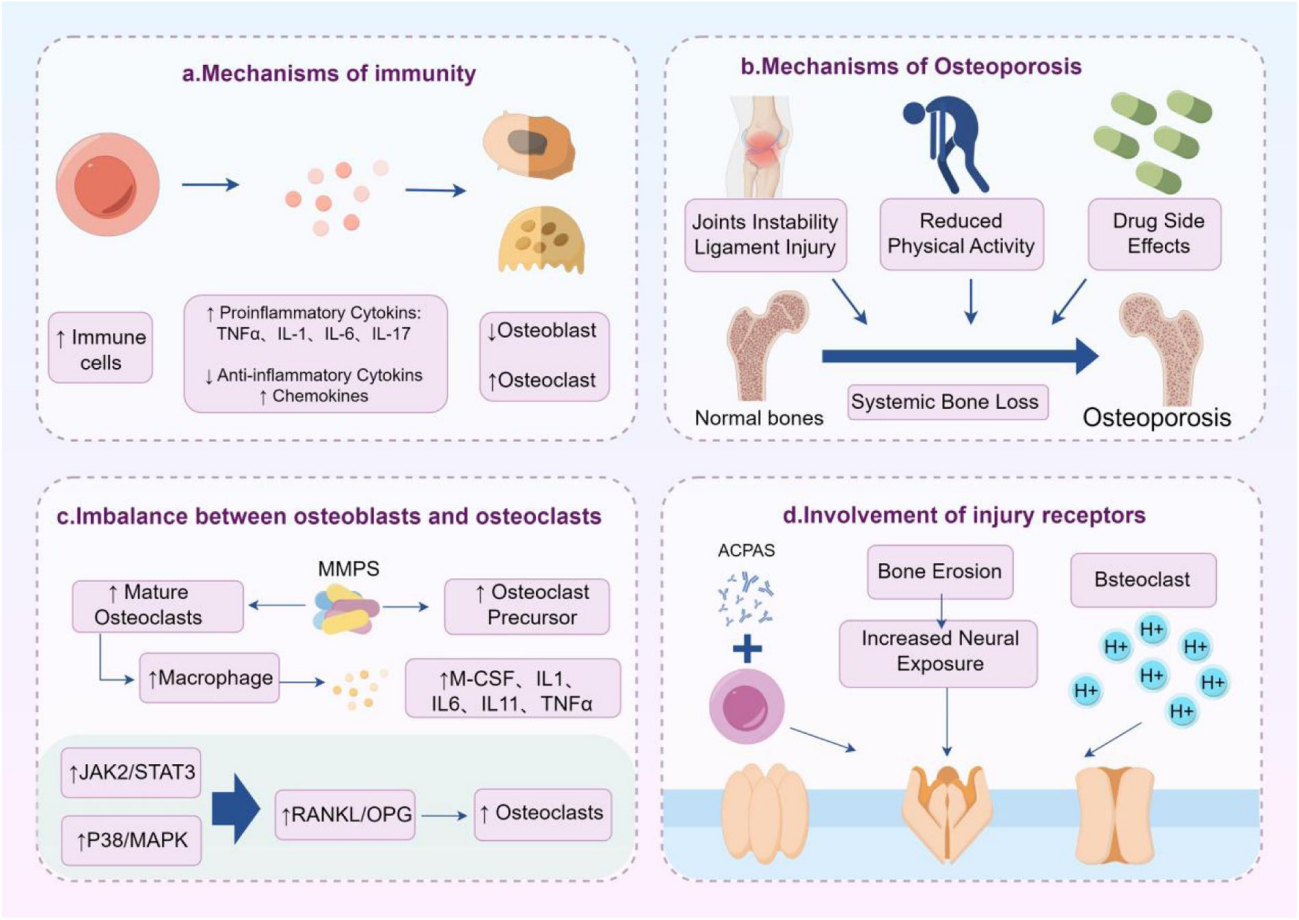
Mechanical pain and structural damage in RA. Inflammation, osteoporosis, imbalance between osteoblasts and osteoclasts, and activation of nociceptors are important mechanisms of mechanical pain in RA.

Mechanical pain is one of the most common forms of pain in RA and is currently the most concerning pain behavioral indicator in animal models. In late RA, joint destruction such as cartilage erosion and osteophyte formation can lead to mechanical pressure pain. Synovitis and joint structural damage are the fundamental causes of mechanical pain, and some key ion channels have been reported to be involved in abnormal mechanical pain in RA. Piezo1/2, TRPV4, ASICs, and other mechanosensitive channels are significantly upregulated or functionally enhanced in inflammatory environments and convert mechanical stimuli into electrical signals. Piezo channels can respond to pressure changes, and TRPV1 and TRPV4 can be activated by both inflammatory factors and mechanical forces (123, 124, 156). Research has confirmed that TRPV1 and ASIC3 are involved in abnormal mechanical pain in CIA, leading to increased expression of ASIC3 on nerves supplying blood to the joint. At the same time, TRPV1 expression in DRG is also upregulated. Compared with the CIA group, the treatment group showed reduced proliferation of synovial cells, leukocyte infiltration, and cartilage destruction. The treatment group produced anti-inflammatory and analgesic effects by inhibiting the activity of TRPV1 and ASIC3. Therefore, many selective and non-selective ASIC3 and TRPV1 inhibitors have shown the potential to alleviate pain and inflammation in animal RA models (157, 158) (Figure 3).

## 6 Psychological factors and central regulation

Anti-inflammatory and analgesic measures play an indispensable role in relieving pain in most RA patients, but some clinical-level pain is still mentioned which is highly likely related to psychological factors (159). The incidence of negative emotions such as anxiety, depression, and anger in RA patients is as high as 50% (160, 161), which poses significant challenges to simple drug therapy. Although the biological mechanisms by which negative emotions mediate RA pain are not clear, it is crucial to acknowledge the important role of negative emotions in RA. There is a bidirectional regulatory effect between emotions and RA pain. On the one hand, the persistent pain and fatigue of RA may trigger depression, anxiety, and even posttraumatic stress disorder (PTSD). Chronic pain triggers anxiety, depression, enhancing pain perception through the anterior cingulate cortex and amygdala, forming a vicious cycle of “pain emotional deterioration inflammation exacerbation” (162, 163). Secondly, joint deformities and limited mobility may cause patients to lose independence, work ability, or social opportunities, leading to serious psychological problems such as an inferiority complex and loneliness. Simultaneous treatment with medication may directly lead to emotional fluctuations, insomnia, or cognitive decline. On the other hand, long-term stress can activate the sympathetic nervous system and HPA axis, leading to disorders in cortisol secretion. Chronic stress may lead to elevated levels of inflammatory factors such as TNF-α and IL-6, exacerbating the inflammatory response in RA. Negative emotions such as depression and anxiety may reduce patients’ tolerance to pain and amplify pain perception, and the degree of pain is related to emotional intensity (164). Emotional disorders further lead to patients neglecting treatment such as missing a dose of medication, reducing physical activity, or deteriorating sleep quality, indirectly exacerbating the condition. Insufficient social support, feelings of loneliness, or negative perceptions of illness can affect the function of the immune system, leading to poor drug treatment outcomes. A clinical report shows that one in every five RA patients suffers from clinically significant anxiety and depression, 26% of RA patients suffer from subclinical anxiety and depression, and the degree of depression and anxiety is positively correlated with the severity of pain (165, 166). The occurrence of depression and anxiety is mainly related to congenital immune system abnormalities (160). The mutual influence between RA and emotional disorders may be due to their common pathogenesis, mainly including proinflammatory mechanisms such as TNF-α; interferon-α, IL-1β, IL-6, and IL-17; oxidative stress factors, and neurotransmitter changes at the CNS and blood–brain barrier (BBB) cell levels (25, 167). Multiple studies have shown that psychological intervention can significantly improve pain scores and quality of life in RA patients, but the direct impact of emotions on RA is still controversial, which may be related to individual genetic susceptibility, disease stage, and other factors.

## 7 Treatment strategies and targets

At present, there are some management strategies for RA pain in clinical practice, including drug and non-drug methods. Drug therapy is the main and key way to alleviate RA pain, including NSAIDs, GCs, traditional antirheumatic drugs (methotrexate, leflunomide, sulfasalazine), biologics, and targeted synthetic DMARDs. Biological (targeting the cytokine mechanisms discussed in Section 2.2) agents are one of the most commonly used drugs in clinical practice, such as TNF-α inhibitors (adalimumab), IL-6 inhibitors (tocilizumab), and B-cell targeting (rituximab). They relieve pain by inhibiting inflammation and are commonly used for poor response to traditional DMARDs or moderate to severe active RA pain. Targeted synthesis of DMARDs is currently the hottest drug, such as JAK inhibitors (tofacitinib, baritinib) and NGF inhibitors (used in Section 3.3) (168). Studies have shown that tandolumab monoclonal antibody has shown significant analgesic effects in clinical trials (169). Targeted synthesis of DMARDs is convenient for oral administration, fast acting, and inhibits intracellular inflammatory signaling pathways, making it suitable for patients with drug resistance to biologics. If RA patients have severe pain, they can try gabapentin, pregabalin, tricyclic antidepressants (amitriptyline), and other drugs or topical NSAIDs gel such as diclofenac gel or patches (170) (targeting the RA pain and neuroimmune interaction mechanisms discussed in Section 4). In addition, complement inhibitors such as anti-C5a drugs have also entered the clinical validation stage. Non-pharmacological treatments include physical therapy and rehabilitation, surgical treatment, and psychological intervention (19). Therapeutic exercises, including low-intensity aerobic exercise (swimming, cycling) and joint range of motion training, are recommended. RA patients with severe joint deformities or functional loss require synovectomy, joint replacement surgery (hip and knee joints), and tendon repair surgery (hand function reconstruction) (171). At the same time, combining cognitive behavioral therapy to provide psychological counseling for patients with chronic pain often accompanied by anxiety and depression (targeting the mechanisms of psychological factors discussed in Section 7). In addition, although in the experimental stage, some emerging therapies and research directions have also been proposed, such as stem cell therapy, new biological agents, and personalized medicine. New drugs have shown good application prospects in the treatment of RA and are currently under active research and development.

## 8 Summary and discussion

**Figure 4:**
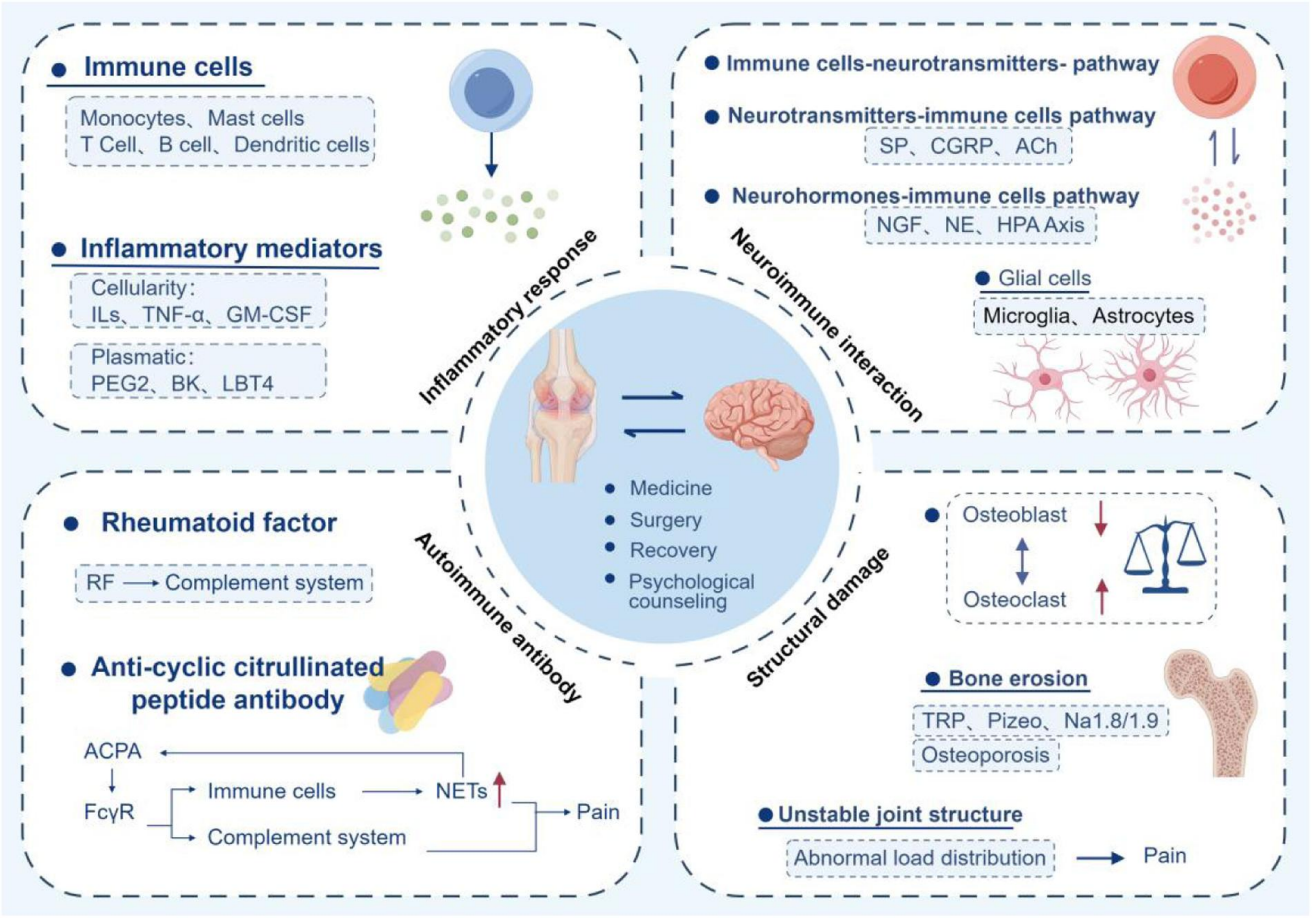
Conceptual diagram and treatment methods of the mechanism of RA pain. Inflammation, autoantibodies, neuroimmune interaction, structural damage, and psychological factors are key mechanisms. Medication, surgery, rehabilitation, and psychotherapy are effective treatment methods.

Pain is an undeniable symptom of RA and one of the indicators of patient satisfaction with RA management. The exploration of pain mechanisms is the primary step in managing RA pain. The pain mechanism of RA is multifaceted and multilevel, including inflammatory response, neuroimmune interaction, autoimmune antibodies, bone structure damage, and psychological factors. This review emphasizes the complex pain mechanisms in RA and targeted pain management strategies. The multidimensional mechanism of RA pain requires a comprehensive treatment strategy that combines anti-inflammatory, immune regulatory, and neural regulatory measures to optimize patients' quality of life. The pain management of RA requires a multimodal and tiered strategy, combined with drug and non-drug interventions, and early intensified treatment to delay joint injury. Patient education, regular follow-up, and psychological support are key to improving quality of life. The treatment plan should be dynamically adjusted based on disease activity, comorbidities, and patient preferences, ultimately achieving disease control and functional improvement (Figure 4).

Although there is currently some research and evidence on the pain mechanism of RA, the pain mechanism of RA is not completely clear, and there are still some unresolved issues and research directions. Firstly, the specific interaction mechanism between cytokines and neuronal receptors is not very clear. Inflammatory factors are deeply involved in RA pain, but do different inflammatory factors, such as IL-1 β and IL-17, have different amplification effects on pain signals? For example, IL-17 may release PGE2 by activating astrocytes, but its dose-dependent effect has not been quantified yet (172). Secondly, in peripheral sensitization, the correlation between paracrine signals between immune cells in the synovitis microenvironment and DRG neurons and dynamic changes in the course of the disease is unclear (173). Single-cell sequencing of RA patients showed an increase in the proportion of IL-17R^+^ neurons in DRG, but its functional validation is still insufficient. The specific molecular pathways of central sensitization in RA are also unclear. How do IL-1β and TNF-α released by microglia activate spinal cord neurons (174)? Recent studies have found that the increased activity of caspase-1 in the spinal cord of RA patients is associated with the severity of pain, but the specific activation threshold is still unclear. Research suggests that the release of CGRP and fractalkine (FKN) in the spinal cord may be involved in this process, but their regulatory network still needs further exploration. As an important link in RA pain, the evidence for the direct analgesic effect of ACPAs needs further verification. Therefore, research on the mechanism of RA pain still needs to be continuously explored, and the management of RA pain requires interdisciplinary collaboration from the laboratory to clinical practice.
